# Radiology and Enterprise Medical Imaging Extensions (REMIX)

**DOI:** 10.1007/s10278-017-0010-6

**Published:** 2017-08-24

**Authors:** Barbaros S. Erdal, Luciano M. Prevedello, Songyue Qian, Mutlu Demirer, Kevin Little, John Ryu, Thomas O’Donnell, Richard D. White

**Affiliations:** 1 Radiology Department, The Ohio State University Wexner Medical Center, 395 W 12th Ave, Columbus, OH 43210 USA; 2 Siemens Medical Solutions USA, Inc, 40 Liberty Boulevard, Malvern, PA 19355 USA

**Keywords:** Enterprise medical imaging, Image reconstruction, Quantitative imaging, Business intelligence, Artificial intelligence

## Abstract

Radiology and Enterprise Medical Imaging Extensions (REMIX) is a platform originally designed to both support the medical imaging-driven clinical and clinical research operational needs of Department of Radiology of The Ohio State University Wexner Medical Center. REMIX accommodates the storage and handling of “big imaging data,” as needed for large multi-disciplinary cancer-focused programs. The evolving REMIX platform contains an array of integrated tools/software packages for the following: (1) server and storage management; (2) image reconstruction; (3) digital pathology; (4) de-identification; (5) business intelligence; (6) texture analysis; and (7) artificial intelligence. These capabilities, along with documentation and guidance, explaining how to interact with a commercial system (e.g., PACS, EHR, commercial database) that currently exists in clinical environments, are to be made freely available.

## Introduction

Within academic medical centers, large sets of data are continuously processed in clinical workflows. These transactional data include patient information collected during routine clinical care or clinical research. Such data can be any, or a combination, of the following: (1) structured/categorical data; (2) semi-structured or unstructured free-text data; or (3) pixel data from radiologic or visible light-based sources. Depending on its nature, the collected data can be stored within: (1) hospital information systems (HIS); (2) radiology information systems (RIS); (3) picture archival and communication systems (PACS); (4) Vendor Neutral Archives (VNA); or (5) computers directly attached to imaging systems (e.g., CT scanners). The collected data can be made available for the following: (1) real-time access (in most clinical situations); (2) near-time access (in most operational and administrative instances); or (3) delayed access (such as for business operations, quality analysis, research, and education). In most cases, delayed access is inherently limited with clinical systems (e.g., HIS, PACS), which emphasize transactions and facilitate immediate availability of patient records. While some data are stored indefinitely for regulatory and legal reasons, most others (especially image data) are discarded once representative subsets are extracted. For example, raw image data from a CT examination are typically discarded once the reconstructions fitting the clinical indication have been created and stored in PACS.

For the majority of clinical systems, application program interfaces (APIs) and back-end databases are, in general, tuned and indexed for real-time access. This is usually done in order to prevent unnecessary stress on clinical/transactional system resources from queries for bulk retrievals of any data type (structured/categorical, free-text, or pixel). Nevertheless, the need to recall and/or aggregate data from large datasets can be justified for a variety of important reasons (e.g., Business Intelligence (BI)). In order to address such growing needs in our own institution, we introduced hardware/software modules that have greatly facilitated essential data collection and processing. This was accomplished by means of a locally developed and evolving program referred to as Radiology and Enterprise Medical Imaging Extensions (REMIX), which was designed to support the medical imaging-driven clinical and clinical-research operational needs of The Ohio State University Wexner Medical Center.

The concept behind REMIX has been to provide a diverse set of tools which can be mixed and matched in an integrated service-oriented fashion. The original REMIX modules/components have been continuously updated based on the appearance of new applications and technologies, while other components have been recently introduced in order to provide distinctly new functionality. While some REMIX components are founded on open-source platforms and packages, others are our adaptation of commercially available hardware/software packages. Our custom code and add-ons made to the vendor-based software, along with any open-source software packages, are being made freely available. This report outlines how REMIX module/packages were intermixed to achieve the desired functionalities of our Department of Radiology, with a focus on quantitative cancer imaging.

### Quantitative Cancer Imaging

For many years, clinical imaging with qualitative interpretation has played a fundamental role in the care of patients with cancers of the blood or soft tissues, positively influencing detection/grading, treatment planning, and/or assessment of therapeutic response on a patient-by-patient basis. From 2000-09, imaging innovation alone is estimated to have reduced cancer mortality by 4% [1]. In recent years, increasing sophistication in earlier and more specific cancer care has demanded greater precision and reproducibility from imaging analyses, with growing emphasis on quantitative imaging [2].

Quantitative imaging represents a range of efforts including the following: (1) standard manual uni-/bi-dimensional measurements of tumor lesion size (e.g., RECIST) on CT or MRI [3]; (2) multi-dimensional imaging with standard manual measurements of tumor size: (a) replaced by completely/semi-automatic volumetric lesion segmentations [4]; (b) complemented by measures of tumor heterogeneity by addition of the dimension of regional tissue signal variability [5]; or (c) complemented by measures of tumor vascularity by addition of the dimension of contrast density/intensity arterial-phase changes (e.g., Choi criteria) on CT or MRI [6–8]; and (3) functional imaging with quantitation of tumor activity based on metabolic profiling on MR spectroscopy [9], tumor utilization of injected labeled metabolites (e.g., PERSIST) on PET [10], and water molecule composition/mobility on MRI [11]. New MRI concepts provide the potential to noninvasively quantify multiple important tissue properties simultaneously through new approaches (e.g., MR fingerprinting) to data acquisition, post-processing, and visualization, thus allowing specific characterization of a target tumor [12].

The term “radiomics” refers to the high-throughput extraction and analysis of large amounts of advanced quantitative imaging parameters from standard-of-care medical images [13, 14]; radiomics data are in a mineable form that can be used to build descriptive and predictive models relating image features to tumor phenotypes or gene-protein signatures [13, 15].

### Current and Past Large-Scale Quantitative Cancer Imaging Projects

The Quantitative Imaging Network (QIN) supported by the National Cancer Institute (NCI) is designed to promote radiomics-related research, along with development of quantitative imaging methods and candidate biomarkers for measurement of tumor response in clinical trial settings [16, 17]. The QIN has demonstrated, through its leveraging of The Cancer Imaging Archive (TCIA) [18], that sharing of standard clinical images across multiple sites for such pursuits is feasible. In addition to DICOM standard clinical imaging data, many TCIA databases provide linked clinical, pathology, and “ground truth” data. Nevertheless, the operations of QIN and TCIA are limited by variability in collections of clinical, imaging, biomarker, and genetic data, as well as reliance on standard-of-care images across its members.

The OPTIMAM Mammography Imaging Database (OMI-DB) contains more than 140,000 breast imaging studies with annotations [19]. While a potentially valuable data source for machine-learning algorithm development for breast imaging, data access is limited by the fact that researchers must follow an application process and submissions need to be reviewed by a steering committee.

There have been earlier attempts in building radiomics hardware/software infrastructures through efforts such cancer Biomedical Informatics Grid (CaBIG) [20]. While including support for collecting images through web-service-based applications, such as caGrid [21], these efforts were eventually discontinued due to large-scale software development costs associated with the project.

Informatics for Integrating Biology and the Bedside (i2b2) [22] is a system allowing institutions can mine their own data through normalized data models. However, it currently does not support processing of imaging datasets.

### ORIEN Project

**Fig. 1:**
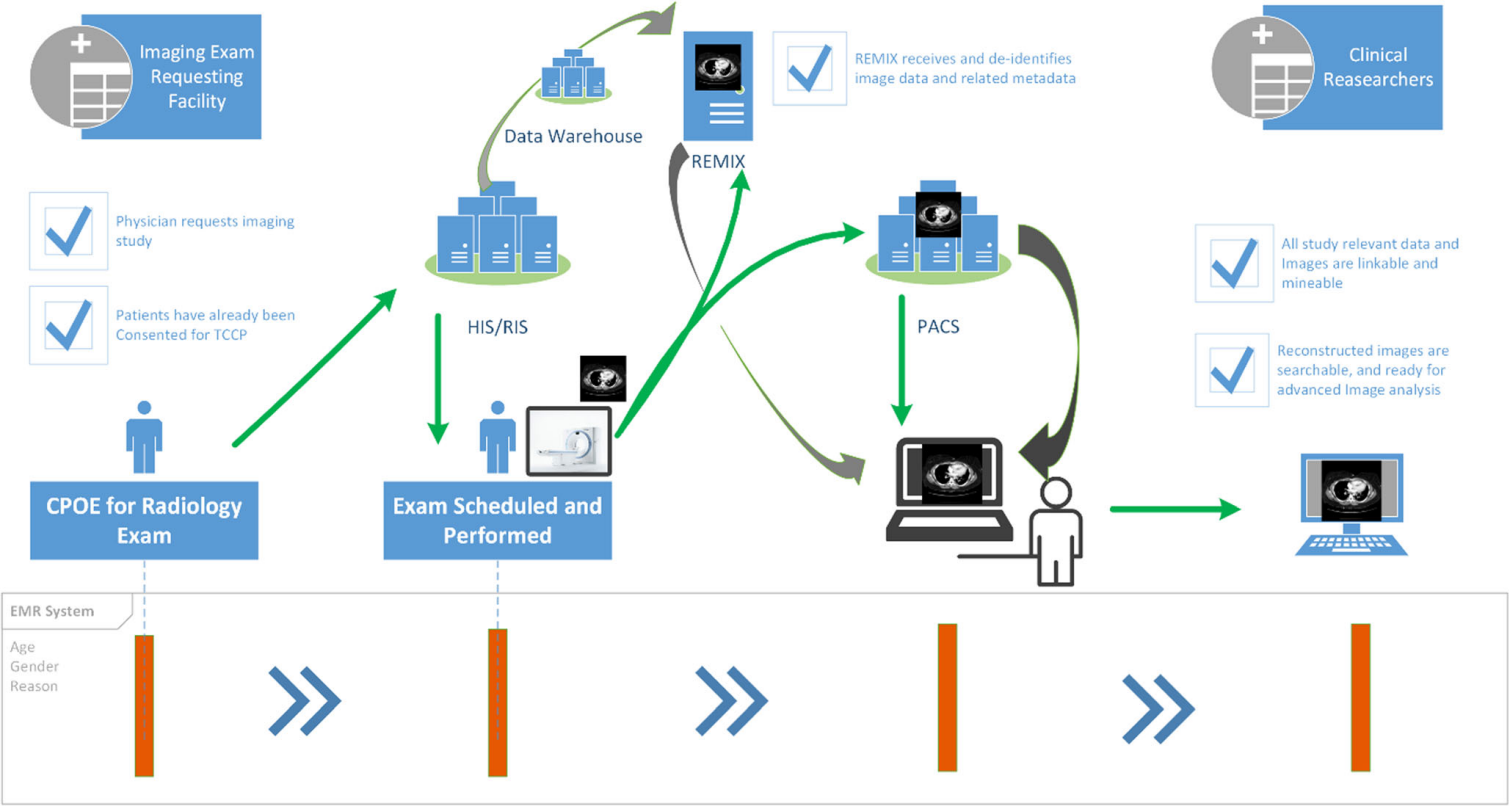
Use of REMIX to address core functionality needs of ORIEN. Collecting/serving and processing data within clinical environments with minimal impact

Initial design principles for REMIX (core functionalities) were guided by our original institutional role in addressing the image-driven needs of the Oncology Research Information Exchange Network (ORIEN) [23]. REMIX [24], at its core, is designed to accommodate the storage and handling of “big imaging data,” for large multi-disciplinary cancer-focused programs, including ORIEN and The Cancer Moonshot project [25, 26]. To this end, REMIX was originally constructed of multiple modules (Fig. 1). In order to provide a more user-friendly and familiar environment, we utilized vendor-based and FDA-approved tools, where applicable, for compatibility with clinical research environments in the future. While initial developments were designed for solving issues related to ORIEN, modules were added later to support anticipated future needs for BI and artificial intelligence (AI) functionalities, as we experience growing needs within our own local operations.

The remainder of this report describes the planning and design of the evolving REMIX platform to this point in time.

## Materials and Methods

**Fig. 2:**
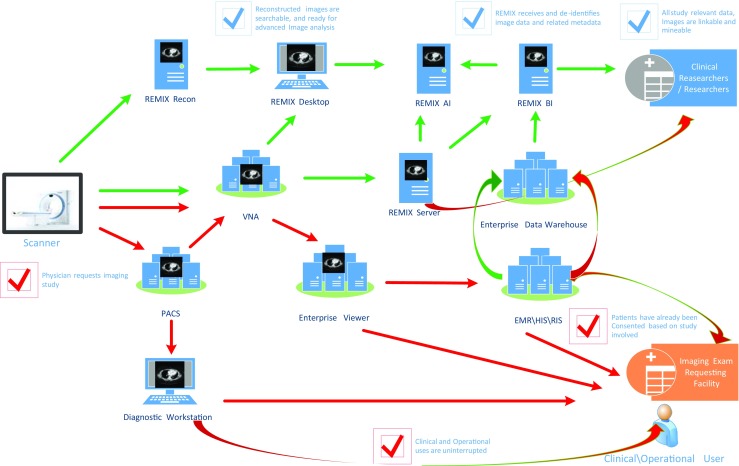
REMIX as an enterprise level platform/framework to address imaging related needs for research as well as operations. *Red lines* operational data flow and *Green lines* research data flow

As one of the more complex clinical data types, imaging-related data can take many forms. Currently, the handling of routine transactional data to support regular clinical and clinical-research operations of our department of radiology is reasonably well-defined using current data standards (e.g., HL7, DICOM, ICD-10). Consequently, we adhered to these standards, as much as possible, while building our REMIX modules in order to provide easier integration (Fig. 2). In some areas (e.g., digital pathology) where there is both considerable vendor dependency and variability in application of nonstandard formats, we tried to follow web standards for displaying and/or managing content for easier integration.

Once deployed and connected, the communication between modules can now be handled in multiple ways, such as the following: (1) web services; (2) RESTful API calls; (3) SQL queries; (4) DICOM; (5) HL7 messages; and (6) custom Python scripts. These methods will be described further for each module as they currently exist; as modules are publically released, further module-specific documentation will cover details on installation and usage.

At this time, the REMIX platform includes the following main components:

### Server and Storage Components (“REMIX Server”)

Currently, the REMIX Server runs in a VMware 5.5 [27] environment on a blade with specifications depicted in Table 1. Depending on usage levels, the storage functionality of the REMIX Server can support interactions with storage area network (SAN) and/or cloud-based storage systems.

**Table 1:** Current hardware specifications for REMIX Server

Manufacturer\model	HP ProLiant BL 465c G7
CPU cores	24 CPUs × 2.399 GHz
Processor type	AMD Opteron™ Processor 6234
Processor slots	2
Cores per socket	12
Logical processors	24
Number of NICs	8

Based on this configuration, a typical VM module deployed (e.g., REMIX De-ID described next) can have specifications such as the following: 4 Virtual CPUs, 8GB RAM, 125GB primary disk, 200GB primary, VMXNET3 network interface and VMware default video interface. When larger storage resources are needed, storage from our SAN is mounted. The REMIX Server is managed by our Division of Medical Imaging Informatics (DMMI), Department of Radiology.

### Data De-identification Components of REMIX Server (“REMIX De-ID”)

Based on a Radiological Society of North America (RSNA) Clinical Trials Processor (CTP) adaptation [28], REMIX De-ID enables image datasets to be de-identified and/or anonymized. It is made available through a custom web interface, allowing data custodians or honest brokers (HBs) to process large datasets for clinical research studies. The main enhancements to the RSNA CTP include the following: (1) Metadata for each examination is extracted during processing; while main patient identifiers are saved into structured table fields, the rest of the metadata is saved as an XML datatype, allowing any post de-identification annotations or extracted futures to be tied to the clinical data; (2) image requester information is tracked to enable custom de-identification templates to be applied in clinical trials (a patient may simultaneously participate in multiple trials); (3) a custom web interface to help system administrators control batch processes and their data-send destinations; and (4) REMIX De-ID access from desktop components (e.g., REMIX Desktop described below), within application tabs used during data browsing and downloads to the local system.

In its current form, unless requested by a specific IRB, REMIX De-ID’s default functionality is to remove all secondary capture images and/or screenshots (such as radiation dose pages) from the studies. While it is possible to remove burned-in identifiers based on image coordinates (e.g., patient identifiers on ultrasound images), this is only available as an option rather than as part of default functionality; this was an institutional decision to reduce the risk of accidental exposure of protected health information (PHI) following de-identification. Even though potential PHI leakage is rare for this process, requested image-sets are manually screened afterward de-identification.

Currently, REMIX De-ID is used as the institutional image de-identification tool, processing all institutional clinical trial image datasets. REMIX De-ID currently processes more than 90 K studies per year for researchers and clinicians. When metadata related to an imaging study needs to be kept for future referencing or processing (e.g., during some clinical trials, institutional review board (IRB)-approved research studies), REMIX De-ID utilizes REMIX-BI databases (described below) for metadata mapping and storage. This functionality allows concurrent data de-identification and delivery with or without non-image data being part of the delivery. In the cases where data are to be anonymized, all mappings and links used for data collection are deleted once the data are delivered in order to eliminate trails.

### Research PACS Components of REMIX Server (“REMIX PACS”)

There are two open-source systems available for REMIX PACS: (1) a DCM4CHEE-based module (DBM) [29], where limited long-term storage space is provided to REMIX De-ID on a first-in-first-out (FIFO) basis; and (2) an Orthanc-based module (OBM) [30], which also provides FIFO-limited long-term storage with additional functionalities, such as DICOM Web [31], a RESTful API, and plugins for whole-slide imaging (being tested but not in use in our environment). DBM’s main function is to provide back-end support for REMIX De-ID, while OBM is designed to provide programmatic access, with its REST API, within our environment. OBM can access de-identified datasets from DBM through DICOM query span [32] functionalities. In addition, project-based instances of OBM can be made available, where access to data stored in our VNA is possible. In this case, visibility of images made available for the given project is controlled by the VNA’s folder-based access control mechanisms. OBM already supports active directory-based access control to its instances [32].

### Data Management and Business Intelligence Components (“REMIX BI”)

REMIX BI is one of the most crucial modules and consists of multiple components. It helps to maintain and index all structural/categorical free-text data in our environment, including most image metadata. REMIX BI has been built as an extension to our institutional data warehouse; hence, it has access to data modeled and gathered from many disparate data sources originating from the systems of multiple vendors (e.g., GE Centricity [33], Epic Radiant [34], Siemens [35], coPath [36]). The data extracted, transferred, and loaded (ETL) into REMIX BI has sources that span more than 20 years (some pathology reports date back in time by 37 years) and originate from HIS, RIS, and PACS.

Formulation efforts for our current data model date back to July 2013, during an 18-month effort to convert between RIS vendors (from GE Centricity [33] to Epic Radiant [34] in 2014), we analyzed queries possible to both vendor systems for routine reports and data requests. From review of 486 queries requested in our former environment, as well as queries that would become available in our future environment after conversion, we identified three types: (1) type 1: simple, quick reports needed to run on HIS and RIS to facilitate operational workflow (e.g., “Find all studies that have not been sent to PACS from the scanners today”); (2) type 2: longer-term reports which either still needed to be accessed from clinical interfaces (e.g., weekly patient schedules, tracking of various order types and events) or involved patient tracking as part of the clinical workflow (e.g., MQSA reports [37]); and (3) type 3: long-term reports where the query dates spanned months or years (e.g., quality or financial analyses, research-related queries).

### Traditional Data Warehouse Component (of REMIX BI)

With the implementation of our new RIS platform in 2014, we made type 1 and type 2 queries available to end-users of HIS/RIS as part of the clinical and operational environment. Along with a one-time historical data ETL, we moved all data collected up to that point into a dimensional data model [38] accommodating both old and new data. This new dimensional model also facilitated the aforementioned three query types with a 1-day lag time (ETL to this dimensional model runs daily, but with the schedule modifiable if more or less frequent updates are needed). This approach also enabled tracking of all events during our RIS exchange and prevented many mistakes during system conversion.

This component of the REMIX BI runs on Oracle Business Intelligence Enterprise Edition (OBIEE) [39], and it is accessed on a daily basis by radiologists, lead technologists, and administrators within our healthcare system. For example, this module can be used by the following: (1) a radiologist in order to track his/her own productivity (e.g., total wRVUs, average reporting turn-around times) on a near-real time basis; (2) a lead technologist in order to track and report on various quality and safety metrics; or (3) an administrator in order to track revenue generation. Having the associated data model in sync with other existing data models (e.g., regarding patients, medications, procedures, surgeries) at an encounter and/or order-detail level allows us to also respond to most clinical research-related queries very quickly. For example, it requires less than 10 s to identify a cohort such as “all patients who were diagnosed with lung cancer, and all their non-contrast Chest CT’s from a given scanner.”

### In-Memory, Data-Discovery Component (of REMIX BI)

While the OBIEE-based traditional data warehouse (TDW) component addresses most user queries, it relies on daily ETL processes. In order to give greater flexibility in terms of query types and sources, we have also introduced an in-memory, data-discovery component for queries based on Tableau software [40]. With this enhancement, users can utilize as data sources either the TDW or data sources/tables which are not part of the daily ETL. This allows us to address less-common ad-hoc queries. In addition, if a given query is serving multiple users with similar needs, its sources can be added to ETL of the TDW (for OBIEE access) as well.

**Fig. 3:**
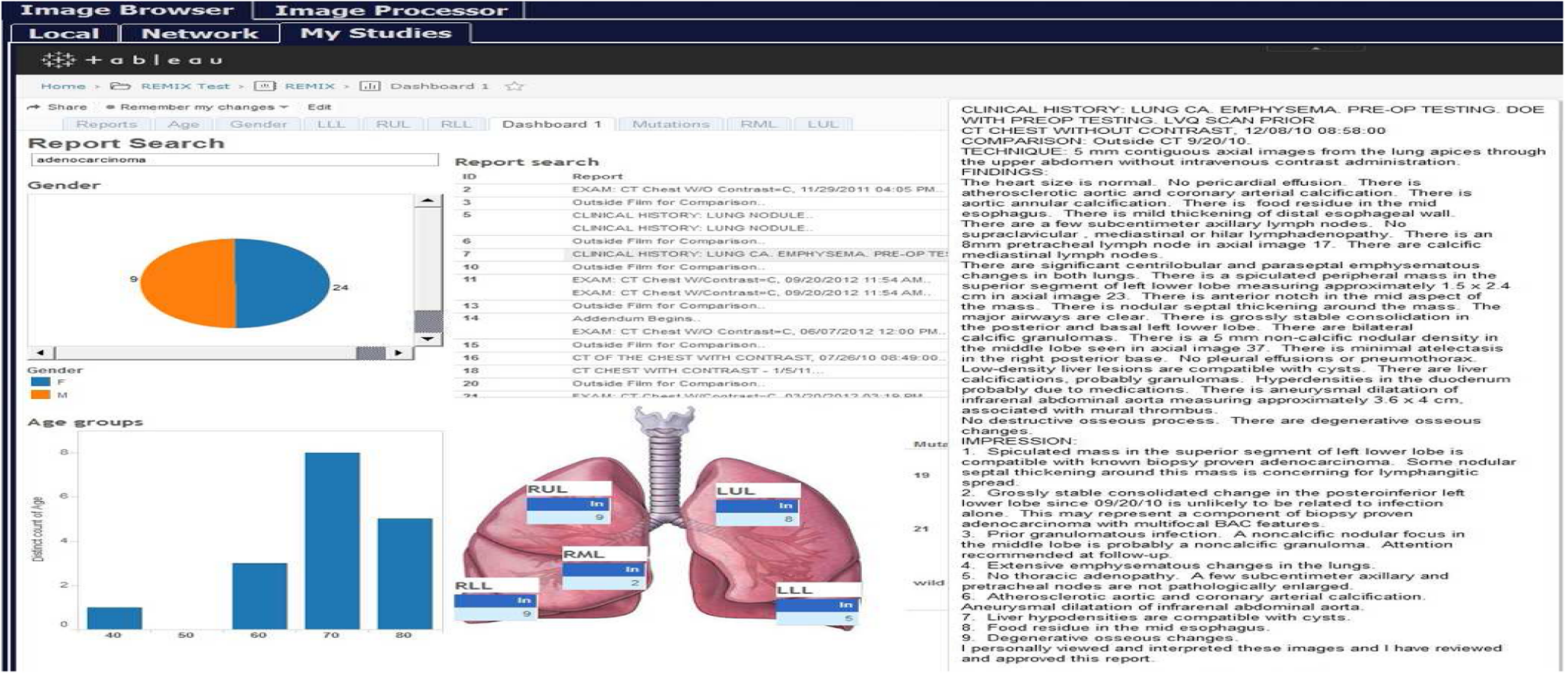
REMIX Business Intelligence (REMIX BI) enables searches on institutional data warehouses as well as custom databases

REMIX BI (Fig. 3) allows users to interface with the entire system using the following: (1) specific modules (e.g., REMIX Desktop described next) from clinical connection points, including the PACS Client and the Electronic Health Record (EHR) and (2) REMIX Web portal (from within REMIX Server). In order to provide data security, concurrency, and continuity within and across institutions, the data management component also provides HIPAA and local IRB-compliant de-identification services. User- and project-based access to folders and views available through REMIX BI is governed by our institutional HB operations committee and the departmental radiology data governance committee.

### Imaging Component (REMIX Desktop)

**Fig. 4:**
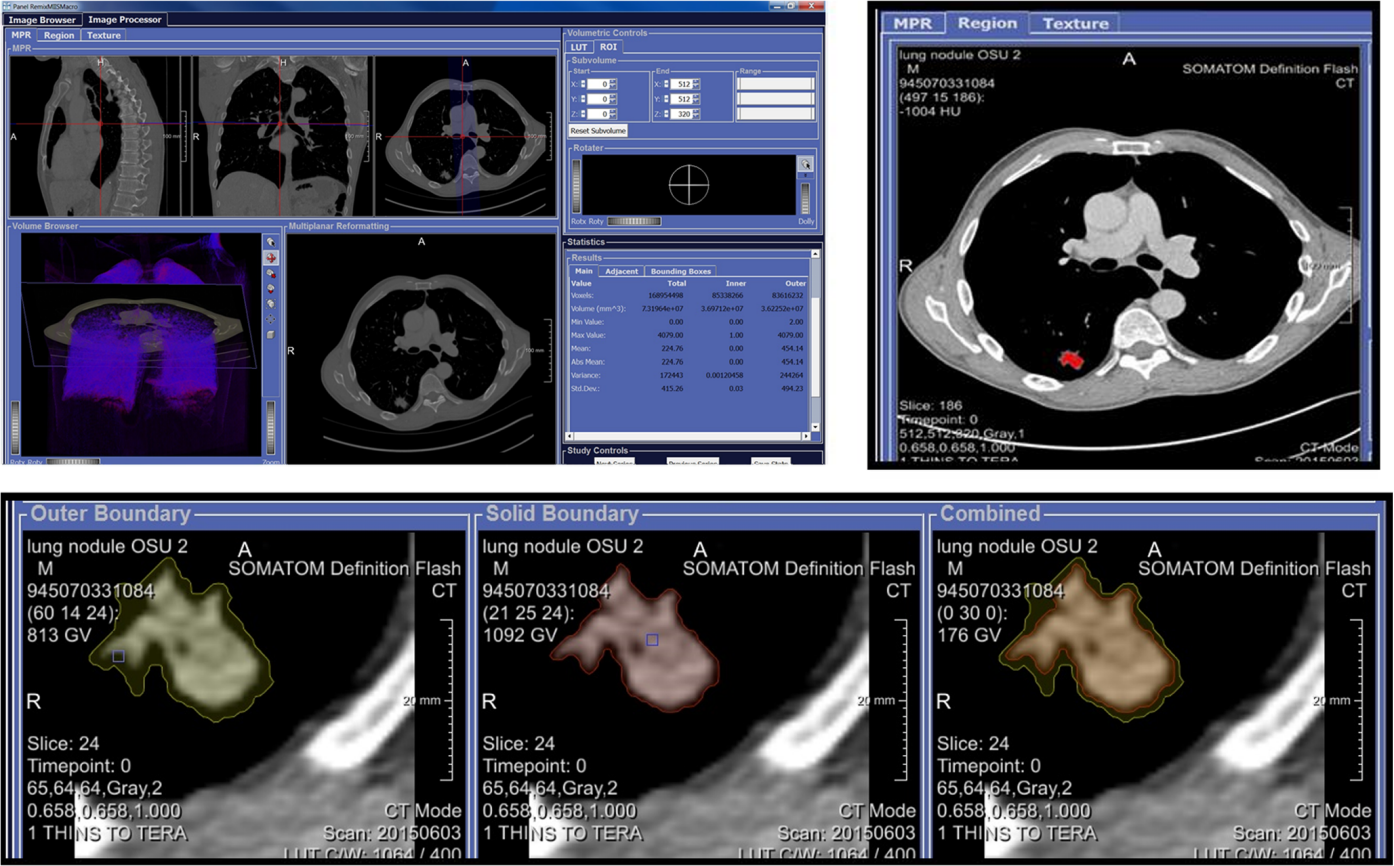
REMIX Desktop for quantitative imaging allows image processing algorithms to be tested and executed on various image types

The REMIX Desktop component (Fig. 4) provides all necessary functionalities for meeting imaging needs, including the following: (1) image storage (through VNA, network storage, and/or local storage capabilities); (2) image segmentation and registration; (3) image viewing; and (4) image processing, including segmentations, histogram-based analysis, and texture-based quantitation. The main functionalities of REMIX Desktop are built upon the MeVisLab programming environment [41], which allows for custom programs/packages to be executed from a single interface. As a result, various custom modules developed as part of REMIX Desktop now include tools for measuring and evaluating lung cancer [42] and interstitial lung diseases [43].

All image-analysis results can be saved into spreadsheets and/or databases for future statistical comparisons or data mining within REMIX BI; such saving to spreadsheets and communicating with commercial databases are functionalities written as custom packages in Python language [44, 45]. Custom SQL queries and database connection capabilities are configuration points within these custom packages. These functionalities enable quantitative image analysis to be linked with patient digital pathology data or genomic data for radiomics- or radiogenomics-based comparisons.

REMIX Desktop also provides a connectivity tab (web interface accessible in an image-browser tab) to REMIX BI and/or REMIX Pathology (described next). Using REMIX BI, databases can be searched for patient cohorts and imaging studies which can be imported for local analysis on REMIX Desktop. Any data de-identification needed at this stage for a research study is provided by REMIX De-ID (web interface accessible in an image browser tab).

### Digital Pathology Component (REMIX Pathology)

**Fig. 5:**
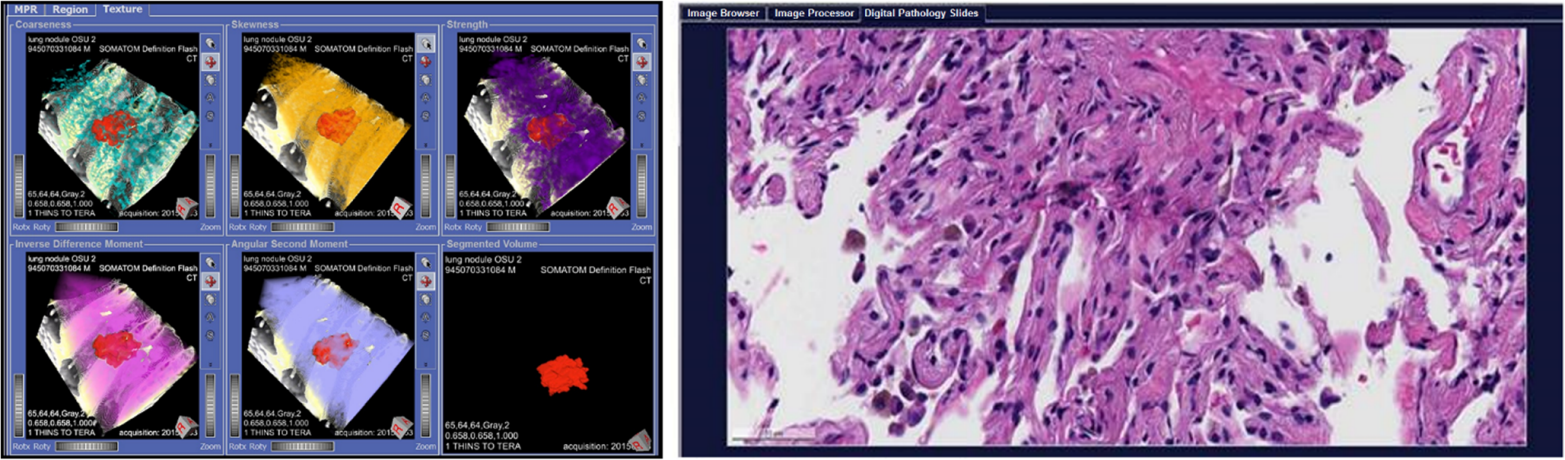
REMIX Desktop for texture analysis and digital pathology

REMIX Pathology is a web-based interface allowing digital pathology images for a given patient to be browsed and displayed that is based on OpenSlide [46]. REMIX Pathology can be used as a stand-alone viewer and/or can be called from modules, such as REMIX Desktop, for viewing relevant pathology whole slides. For example, if users were to investigate an image set for a case of adenocarcinoma of the lung, available digital pathology slides from the biopsy can be viewed concurrently utilizing REMIX Pathology. In its current form, REMIX Pathology (Fig. 5) only serves as a whole-slide viewing component; image processing on digital pathology slides has not yet been included as a functionality of the REMIX platform.

### Image Reconstruction Component (REMIX Recon)

**Fig. 6:**
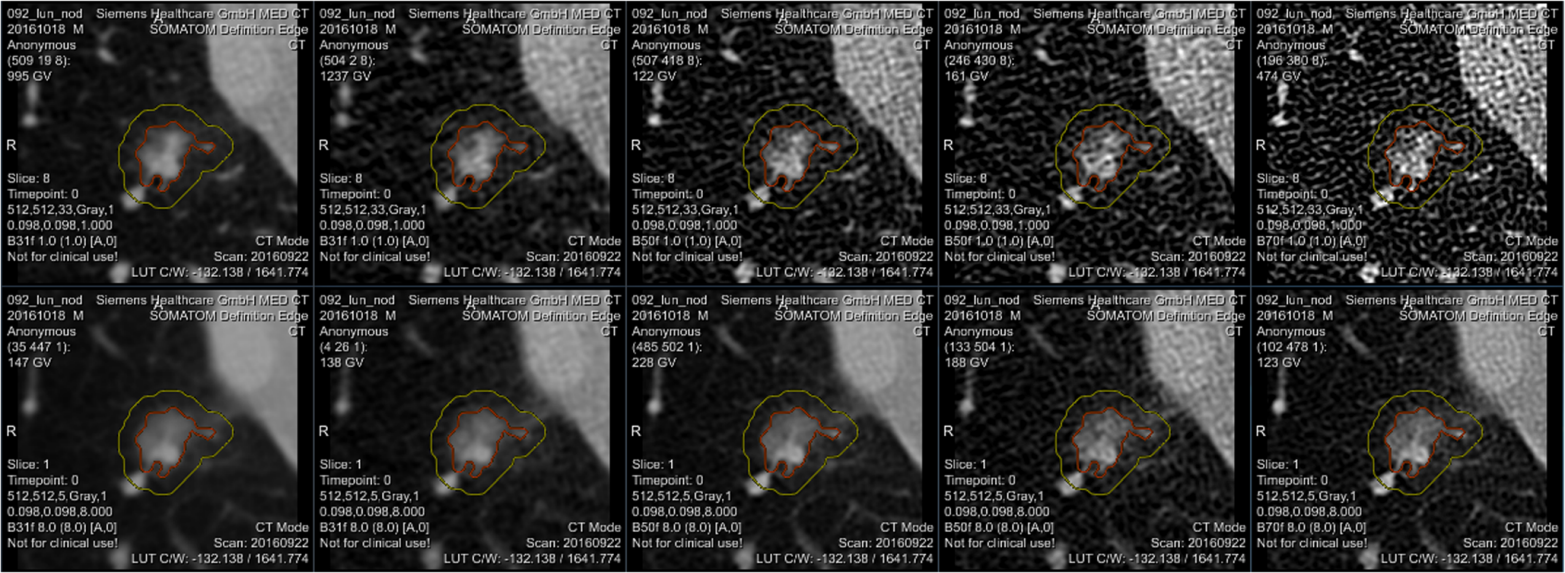
REMIX Recon enables multiple alternative reconstructions to be generated in batch mode

REMIX Recon enables raw image files from the scanners to be reconstructed using varying specifications (Fig. 6). In its current form, REMIX Recon is based on Siemens Recon CT software packages (version 13.8.5.0; Siemens Healthcare, Forchheim, Germany, made available only to our Department of Radiology through a master research agreement with Siemens Healthcare). Images can be produced using the following: (1) different reconstruction algorithms (e.g., filtered-back projection [47] vs. iterative reconstruction methods [48, 49]); (2) various slice-thicknesses; and/or (3) dose-reduction simulations [50–52]. Manipulations can then be performed to identify optimal reconstruction schemes for a given task; this can be a computer-aided diagnosis or AI process. REMIX Desktop can be utilized to assess the influence of these modifications on shape (segmentation), color (gray level), and/or texture.

This module currently processes only CT images; however, source scanners can directly save raw image data into this module’s shared storage. Different reconstructions can be created in batch mode utilizing our custom Python packages, and the resulting reconstructions can be fetched directly into other REMIX Desktop and REMIX AI environments using direct file copies. Developed extensions to the standard RECON CT software include control components (enabling batch modes of the software to be managed, configured, and executed) and other file management properties (enabling interoperability between REMIX Recon and other modules).

### Artificial Intelligence Component (REMIX AI)

**Fig. 7:**
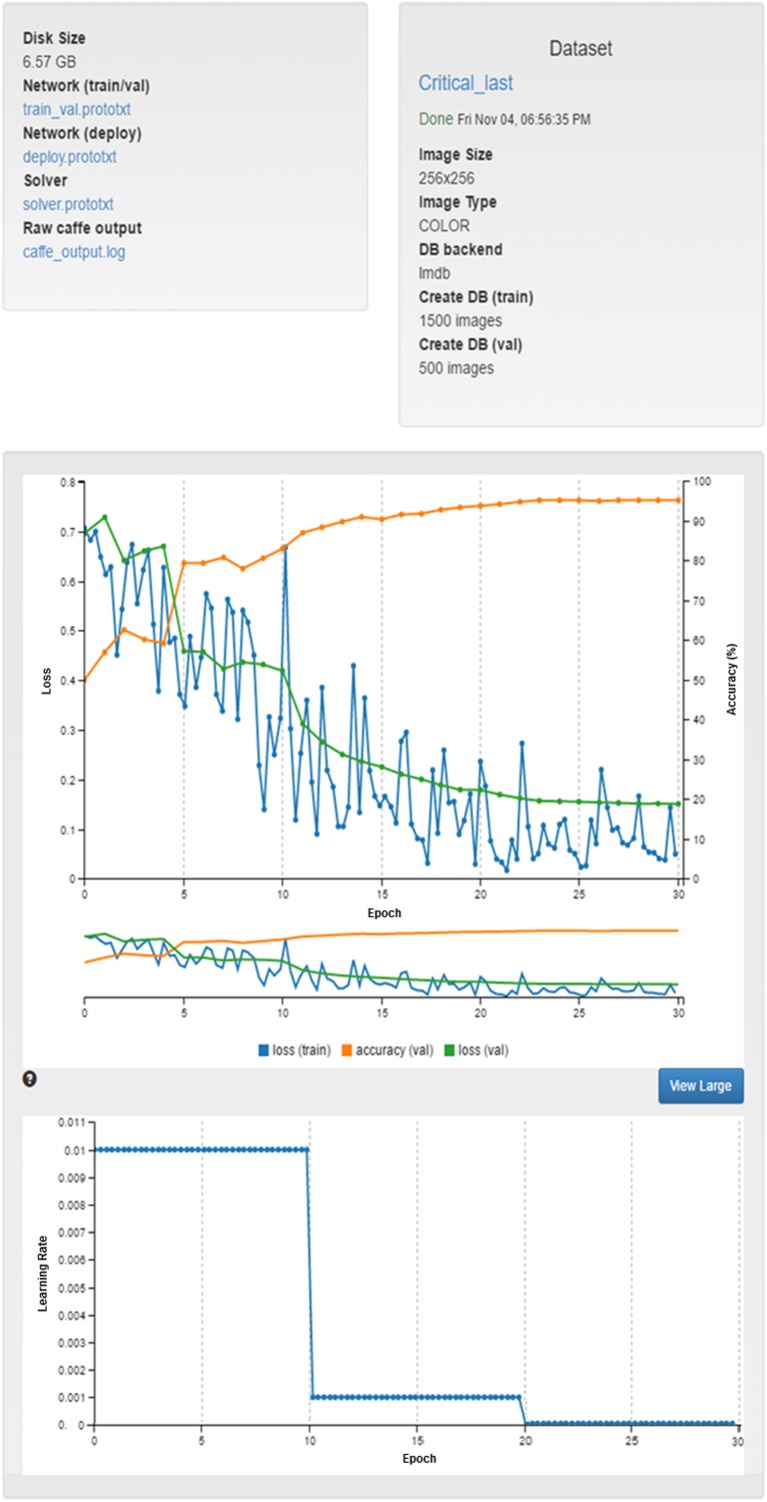
REMIX AI enables multiple deep-leaning algorithms to be executed on image-data collections

REMIX AI enables various machine-learning/deep-learning algorithms [53–55] to be executed on image datasets (Fig. 7). Datasets collected and/or generated through REMIX BI, REMIX Desktop, and/or REMIX Recon can be saved into shared drives; these 2D or 3D image datasets can serve as training cases, test cases, and/or data augmentation cases within REMIX AI. Input/output parameters can be manipulated through a web interface, and the results/progress can be tracked in real-time. The trained neural networks are deliverable to other systems (with similar neural network settings), while maintaining reproducibility [56].

Unlike most of the earlier components which can be deployed as virtual machines, REMIX AI is a dedicated hardware/software solution that is built as an NVIDIA Digits reference system running on Linux Ubuntu 14.04. Table 2 shows specifications of the dedicated-machine learning system.

**Table 2:** REMIX AI hardware specifications

Processor	1 × Intel Core i7-5930 K Processor (15 M Cache, 3.50 GHz)
Memory	64GB DDR4
GPUs	4 × NVIDIA GeForce GTX Titan X GPUs (7 Teraflops of single precision, 336.5GB/s of memory bandwidth, 12GB memory per GPU)
Operating system (OS)	Ubuntu 14.04
Storage	2 × 256GB SSD disk for OS and software libraries and 3x3TB standard disk on RAID 5 for data storage

While AI algorithms, such as GoogLeNet [57] or convolutional neural network frameworks running on Caffe [58], are already available as part of NVIDIA Digits distributions, REMIX AI includes additional libraries, such as PyDICOM [59], NumPy [60], or custom libraries developed for REMIX integration. These Python-based software libraries are for converting inputs from DICOM images to other formats, such as JPEG for color images and/or numerical arrays to be executed on back-end environments like Caffe or Torch [61]. Currently, within NVIDIA Digits-based platforms, image data first needs to be placed into folders for subsequent referencing by respective AI algorithms. Currently, we have three ways for users to load images into REMIX AI by utilizing the following: (1) REMIX AI web interface, allowing users to upload their data into the system; (2) REMIX Desktop, permitting users to directly save their image data into shared disk drives of REMIX AI; or (3) Python-based client libraries, so that users can make RESTful API [62] calls to REMIX PACS.

## Results

The REMIX platform has already had a direct positive impact on streamlining our departmental operations. In this section, we provide some examples of initial use cases along with system performance measurements, where applicable.

### Example 1

This multi-step example shows how the REMIX platform can be utilized for image texture analysis of a case of adenocarcinoma of the lung, as a test of both reproducibility (relative to an earlier study [38]) and improved functionality with this newly developed platform. In the earlier study, lung nodules from 62 non-contrast CT studies were analyzed with Haralick’s textures, and promising results regarding the relationship between texture and malignancy type were reported [38]. Using REMIX capabilities, our aim was to repeat the previous manual processes and to complement them with a more comprehensive analysis in terms of additional texture algorithms accompanied by additional quantitative image reconstructions.

**Fig. 8:**
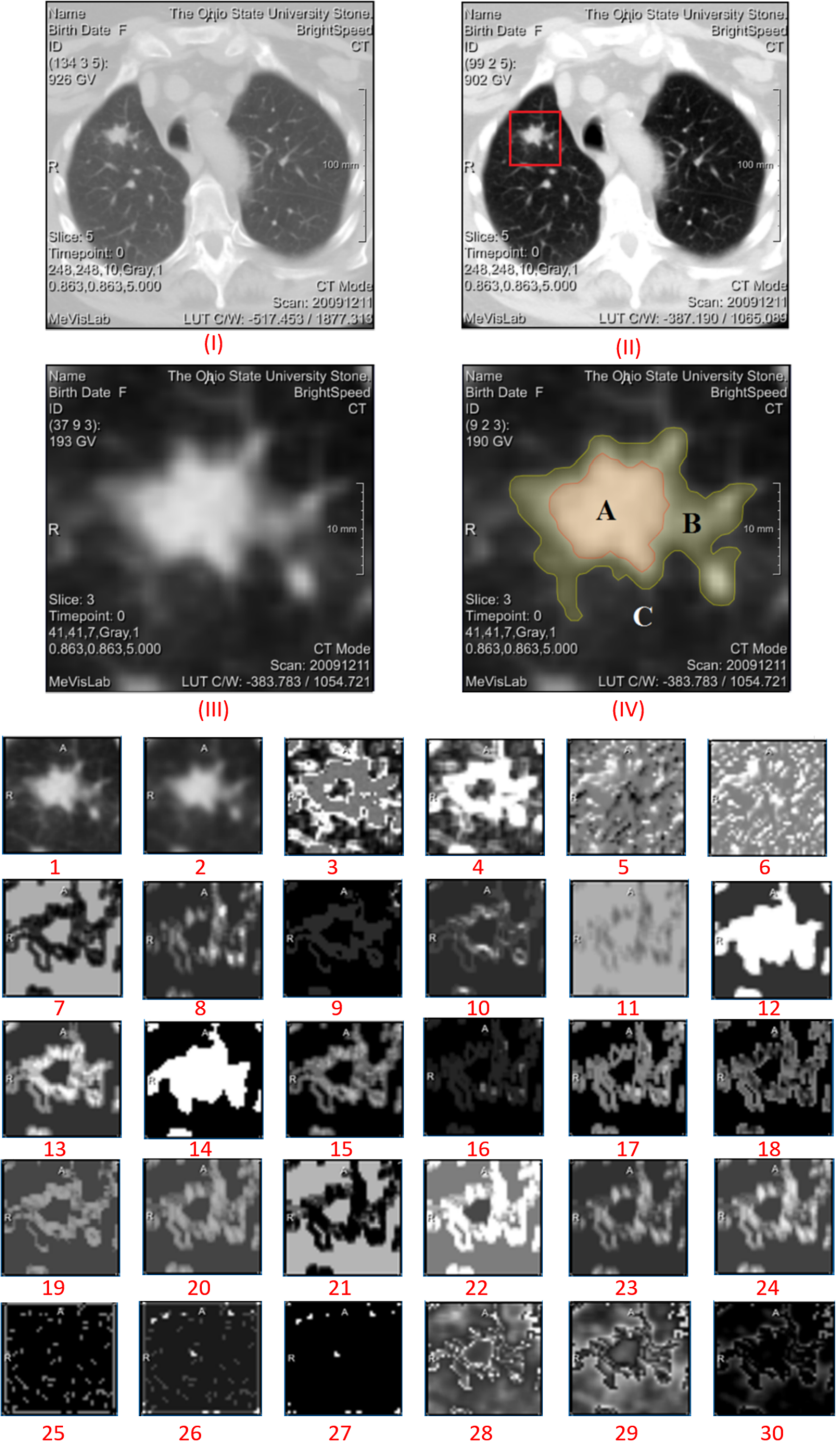
Once an ROI is selected, areas surrounding the nodule can be further sub-segmented: (*I*) original image; (*II*) image after normalization by histogram equalization; (*III*) ROI selected; (*IV*) image subsections. Then for each section, various texture features can be calculated. For this example, we are showing results on the following: original (1); simple average (2); simple contrast (3); simple deviation (4); skewness (5); kurtosis (6); co-occurrence matrix (CCM)-based Homogeneity (7); CCM-based contrast (8); CCM-based correlation (9); CCM-based variance (10); CCM-based inverse difference moment (11); CCM-based sum average (12); CCM-based sum entropy (13); CCM-based sum variance (14); CCM-based entropy (15); CCM-based difference variance (16); CCM-based difference entropy (17); CCM-based measures of correlation 1 (18); CCM-based measures of correlation 2 (19); run-length matrices (RLM)-based short runs emphasis (20); RLM-based long-run emphasis (21); RLM-based gray level non-uniformity (22); RLM-based run-length non-uniformity (23); RLM-based run percentage (24); neighboring gray-level dependence matrix (NGLDM)-based small number emphasis (25); NGLDM-based large number emphasis (26); NGLDM-based second moment (27); neighborhood gray tone difference matrix (NGTDM)-based coarseness (28); NGTDM-based complexity (29); NGTDM-based texture strength (30)

- Step 1: For formulating a cohort utilizing REMIX BI, three queries were applied to form an inner-join dataset (Fig. 3): Query (1) Search for detection of all patients with diagnosis of adenocarcinoma of the lung based on ICD9 and ICD10 diagnosis codes was performed in 2.7 s; Query (2) search for biopsy confirmation of adenocarcinoma from the pathology reports (with free-text searches) was performed in 3.8 s; accompanied with EGFR gene mutation data (Exon 19, 21 mutations, and Wild-Type data); Query (3) search for non-contrast chest CTs prior to biopsy was performed in 2.6 s.
- Step 2: Transfer accession numbers from REMIX BI module to the REMIX De-ID module to facilitate the bulk download, de-identification, and saving of DICOM images to their corresponding destination folders. A total of 66 non-contrast CT image sets, along with associated markers (25 Exon 21, 21 Exon 19, and 20 Wild Type), were identified by step 1. REMIX De-ID was monitored by our DMMI personnel; the download and verification/QA processes required 2 h.
- Step 3: Utilizing REMIX Desktop (installed on desktop system with Intel Xeon E3-1270 v5 @ 3.60GHz, 4 Cores CPU, 32GB system memory, and NVIDIA Quadro K1200 GPU with 4GB graphics memory), DICOM images were processed (Fig. 4). This is an interactive step, where users can browse through volumes. Once a nodule is selected, users can determine the bounding box (region of interest (ROI) size to be included during analysis); for correspondence with the earlier work [38], a 5-cm bounding box was selected. With the earlier work indicating that image futures surrounding the nodule can be utilized during the classification of tumors, REMIX Desktop provided assessments on the following ROI subsections (ROIS): (1) solid components of tumors; (2) nodule and surrounding tissue; (3) surrounding tissue alone; (4) all other tissue within the ROI; and (5) entire ROI, including the nodule and its surroundings. Once selections were made by the end-user, statistics from the different components of the ROI (Fig. 8) were saved into a database or a MS Excel spreadsheet for further analysis. Once trained on the software, it took a radiologist on average 8–20 s to locate the nodule in volume, execute texture analysis within the ROI, and save the results.
- Step 4: Utilizing REMIX Pathology, digital whole slides can be displayed from/within REMIX Desktop (Fig. 5). This is a manual step, where pathology slides from the nodules need to be pre-uploaded to the respective folders. At this stage, REMIX Pathology is utilized only for viewing purposes.

### Validating Results and Beyond

After running this dataset and extracting the statistics on textural analysis, the new results were compared with those from our earlier work with the same dataset [38]. The results were in concordance when comparisons were made metric-by-metric (Haralick’s texture scores of angular second moment, contrast, inverse difference moment, correlation and entropy) [63]. Correlations among the genotypes of nodules and the textures were also demonstrated. For example, Haralick’s contrast was able to differentiate both exon 19 (*p* = 0.00028) and exon 21 (*p* = 0.00001) from the wild type, or inverse difference moment differentiated Exon 19 mutants from Exon 21 mutants (*p* = 0.018).

Utilizing all available metrics within REMIX Desktop with utilization of ROIS (Fig. 8) also enhances our results. For example, applying a Sequential Minimal Optimization [64] classifier (since many more metrics are evaluated), separation of Exon 19 and Exon 21 mutations with 90% (89.5–90.5%, respectively) average accuracy was achieved.

- Step 5: Initial sample sets from the earlier studies were gathered from retrospective data searches and were saved in PACS as 5-mm thick CT images. These results raised the issue of whether thinner slices and/or better control of scanning/reconstruction would improve the accuracy. As mentioned previously, because raw scan files are routinely discarded after a short period of time, a query was formulated using REMIX BI to find all lung cancer patients who were scheduled for non-contrast CT scans on a subset of scanners in our system, where the scanners were already connected with REMIX Recon. On these scanners after a scan is performed, the technologist can directly save a copy of the raw files to REMIX Recon in less than 10 s, without interruption of the daily workflow.
- Step 6: This step is demonstrated on a single case to illustrate how additional reconstructions can be obtained. Utilizing both REMIX Recon and REMIX Desktop, a raw image-file from a non-contrast Chest CT examination demonstrating a lung nodule (Fig. 7) was downloaded in order to assess the impact of varying reconstruction specifications on the produced images. Multiple (*N* = 128) reconstruction versions of each basic image represented permutations in (1) reconstruction algorithm/kernel (filtered back projection/B31, B40, B50, B70; iterative reconstruction/I31, I40, I50, and I70) (*N* = 8); (2) reconstructed thickness (1, 2, 4, and 8 mm) (*N* = 4); and (3) noise level simulating different dose intensity (original scan dose at 100, 50, 25, and 12.5%) (*N* = 4).

Using REMIX Desktop, each version of image reconstruction delineated by a ROI surrounding the lung nodule was subjected to the same analysis. For each ROI, the variation (e.g., mean, median, maximum, standard deviation) in 31 different texture metrics (e.g., GLCM [63], contrast [65]) were monitored. Ultimately, 211 statistical values for five different areas within the ROI (e.g., solid component of nodule, peri-nodule surrounding tissues) were measured; a total of 1055 (i.e., 211 × 5) measurements per reconstruction version were then recorded into both REMIX BI and Excel spreadsheets. This process required approximately 1 min per reconstruction; for this example, the entire duration was 128 min.

**Fig. 9:**
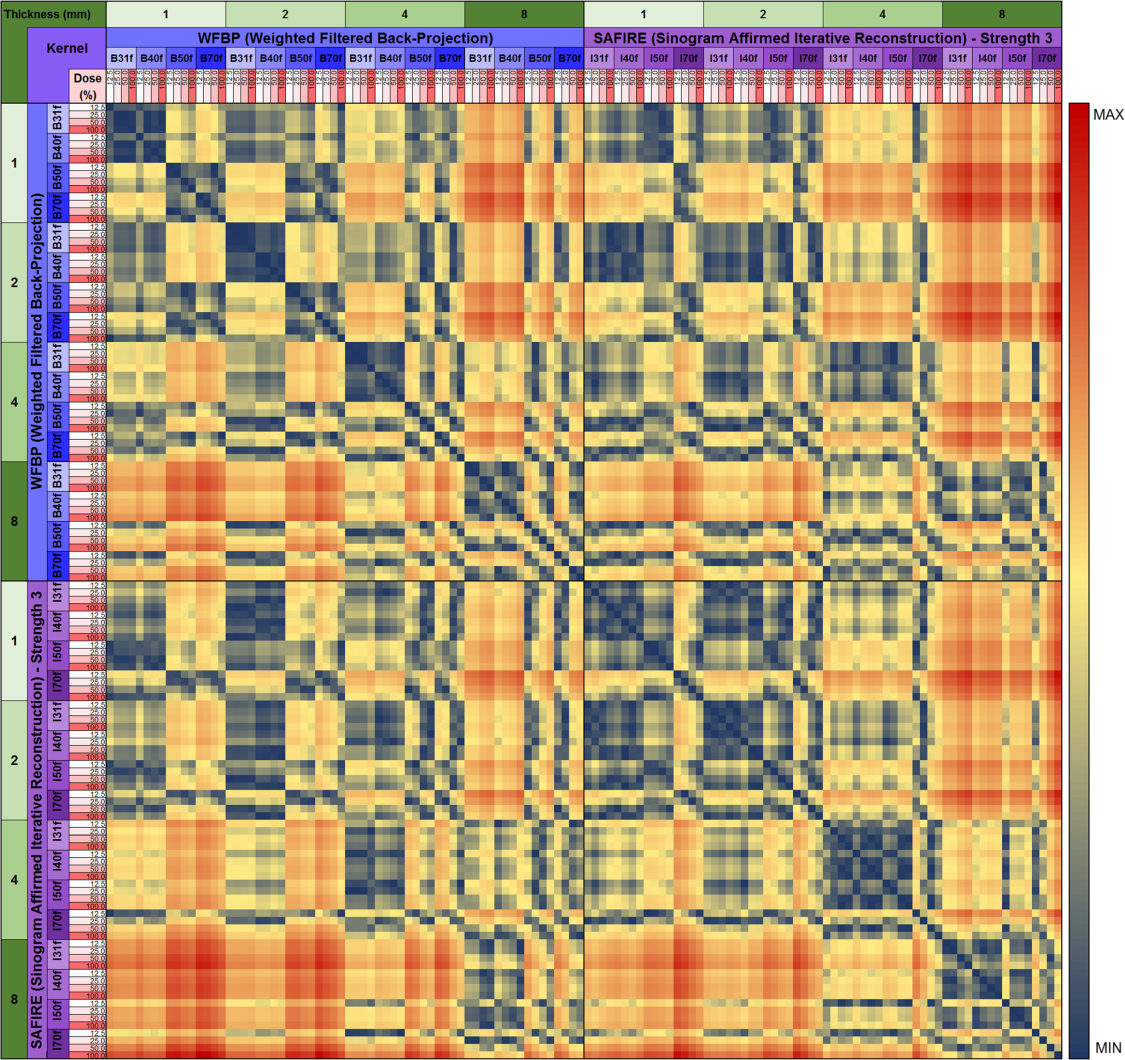
Heat map shows potential effects of varying reconstruction specifications on texture analysis. Changes in a gray-level co-occurrence metric, angular second moment, is given as an example for an ROI containing a lung nodule. The normalized color scale depicts no change in a given metric values as *dark blue* (0), while the maximum change is shown in *dark red* (1)

Early reports of 27 cases with analysis on image reconstructions using REMIX Recon as described in Step 6 indicate potential for significant change in size, gray level, and texture due to differences in reconstruction techniques that could be chosen clinically. Figure 9 shows an example of the effects of varying reconstruction specifications on a given texture metric. Once fully evaluated and verified by a larger number of cases, these results will be separately reported in greater detail.

### Example 2

**Fig. 10:**
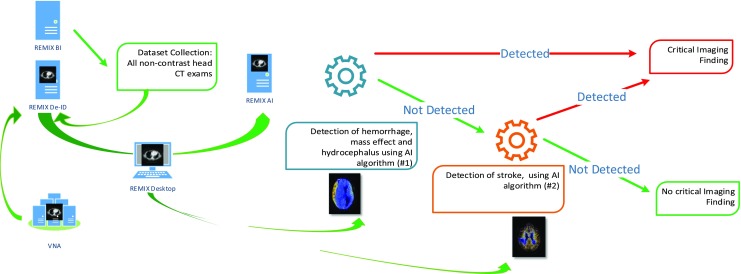
An example AI algorithm deployment strategy within REMIX environment

Utilizing multiple modules, Fig. 10 depicts the utilization of the REMIX platform for AI algorithm training and validation. In this example, we have simulated the steps needed to identify the cohort, collect the appropriate images, and process the dataset for classification of non-contrast head CTs in “critical” versus “noncritical” cases. The results shown here are in contrast to a previously performed manual approach that took several days to be completed, demonstrating the advantage of the integrated REMIX environment. The scientific discussions on the example used here for verification purposes are reported elsewhere [66].

- Step 1: In order to collect the necessary datasets, several queries were executed in REMIX BI to identify the accession numbers for images to be downloaded and de-identified: Query 1: Find all “non-contrast head CT exams” where patients have been associated with “critical findings” (e.g., hemorrhage, mass effect, and hydrocephalus) for a given month; Query 2: Find all “non-contrast Head CT exams” where patients have been associated with “Stroke”. During these initial data discovery steps, we utilized OBIEE interfaces of REMIX BI, with each query returning results under 12 s (Query 1: 11 s and Query 2: 8 s).
- Step 2: Accession numbers were transferred from the REMIX BI module to the REMIX De-ID module to facilitate the bulk download, de-identification, and saving of DICOM images to their respective destination folders (other options included sending to a DICOM AE Title or burning to a DVD); a total of 2583 images were processed for Query 1 and 646 images for Query 2. During this stage, REMIX De-ID was monitored by our DMMI personnel; the download and verification/QA processes took a total of 19 h.
- Step 3: Utilizing REMIX Desktop, DICOM images were converted into color JPEG images using color lookup tables for each respective window and level. During coloring of images originating from Query 1, a “Brain Window” setting was utilized, while coloring images from Query 2 utilized a “Stroke Window’ setting. The results were written into shared folders where they could be accessed by the REMIX AI module. The entire process took 243 min.
- Step 4: In this final step, images were converted using REMIX AI to 256 × 256 matrices and processed with GoogLeNet [57] as the convolutional network running on Caffe [58]. A total of 60 training epochs were used (solver type was Nesterov’s accelerated gradient). The total processing time for model creation with the first dataset (from Query 1, with 2583 images) was 6 min and 19 s, with all four GPUs utilized; for model creation for the second dataset (from Query 2, with 646 images), total processing took 97 s. Once image classification models were created, batch image classifications were completed at a rate of approximately 25 images per second.

### REMIX, Pre-REMIX Time Savings

#### Savings on Searches

With a BI-based search model, in which underlying table joins are already verified and optimized, search times for cohorts decrease from units of days or weeks to seconds. For the original study relating to example 1 [38], it required institutional data warehouse programmers several days to construct SQL queries (processed as research data requests) to identify the patient cohorts. While the actual developer workload was under 8 h (one work day), the validation steps prolonged the query finalization to 3 weeks. However, during the other original study related to example 2 [66], REMIX BI was already operational, saving several weeks for query construction and data searches.

#### Savings on Image Downloads

For both the original studies related to example 1 and example 2, the manual downloading of images required several days. While the RSNA CTP [24] (providing the underlying DICOM functionality for REMIX ID) does not provide any additional efficiencies for image downloading (using DICOM C-Query, DICOM C-Move), REMIX De-ID required only confirming that downloads completed rather than the manual initiation and completion of downloading.

#### Savings on Image Reconstructions

As mentioned in example 1, it required approximately 1 min for each reconstruction to be performed. While a single additional custom reconstruction may not take much of a technologist’s time, reconstructing 128 or 256 reconstructions would consume more than 128 to 256 min of personnel and scanner software time. Hence, rather than spending 128 to 256 min per imaging study, a technologist can forward a copy of raw data in less than a minute to REMIX Recon, saving valuable resources, since REMIX Recon can run in batch mode on its separate software/hardware resource.

#### Savings on Research

Both original research studies related to the aforementioned validation examples relied of retrospective data searches. While example 1 extends several years back in time, example 2 uses 1 month of data (in two separate queries); in both examples, datasets are identified within seconds. It would have taken months and years to collect such data manually if the research studies were carried out prospectively.

In addition, prospective parts of research initiated following example 1, where we look for lung nodules to reconstruct, are also accelerated by looking into patients’ schedules for upcoming chest CT examinations where the patients were already diagnosed with lung cancer. Hence, the search for potential nodules to reconstruct is shortened.

## Discussion

Content-based data mining is of growing interest due to the limitless possibilities to exploit already acquired digital information, such as imaging data, for cancer care. Generation of a common multi-institutional shared imaging (reconstructed and raw)-data repository could support content-based mining for multiple purposes, including the following: (1) promotion of the concept of non-invasive “imaging biopsy” alternatives to invasive tissue sampling based on detection of specific advanced quantifiable image features (from accumulated knowledge and computer-aided diagnosis/AI) of each histologically and genetically profiled tumor type; (2) design of more focused, shorter-duration, and cost-effective prospective studies relying on fully-characterized already available imaging data; and (3) development and testing of new concepts in image reconstruction and quantitative analysis drawing on either reconstructed or raw imaging data. The REMIX platform described in this report supports these needs in an integrated fashion.

Despite the robust technical capabilities of the REMIX platform, there are many data and information security aspects, as well as HIPAA and local IRB regulations, which would need to be considered by an adopting institution. To that end, the following have been locally implemented: (1) institutional HB system exploited by most REMIX components; (2) data-use agreements signed by investigators to ensure adherence with HIPAA and IRB rules supporting PHI security; and (3) trained personnel to continuously monitor and support quality assurance of systems and their outputs.

For organizations seeking to mine their own data, REMIX provides many support mechanisms that are not currently offered by other tools. In addition, it does not impose any specific technology or infrastructure needs such as grid/web services (e.g., caGrid) [20, 21] and allows a modular, standards-based (e.g., DICOM, RESTful services, etc.) deployment. While online data sources such as TCIA [18] and OMI-DB [19] can serve as very valuable, well-curated public data sources, institutions can have access to much larger sets of clinical data if they mine their own data. For example, within our intuition, current annual exam volume is around 600,000 studies, and REMIX BI can search data going back 14 years. This means that a query going back 3 years can already produce a dataset (152,000 mammography studies) comparable to OMI-DB.

There are challenges associated with building large online image databases and/or pulling data from them including the following: (1) The amount of storage needed if all images are to be stored online, and (2) high network traffic, which would potentially be costly. Based on our local cost estimations (for the State of Ohio), this would be a minimum of four to five million dollars of initial setup cost followed by one to 1.5 million dollars per year to host and maintain such datasets. This is based on estimated service for three to four large health systems in the state (estimations were based on two petabyte initial load with half a petabyte increase per year). Unless this was the actual clinical/operational environment (not just for research), it would be hard to justify such expense. This leaves the option to extract such datasets as requested from the clinical systems as efficiently as possible with minimal or no impact on daily clinical operations. As mentioned previously, clinical systems are usually transactional systems; hence, it is crucial to first identify data to be pulled from them and later to pull the data as efficiently as possible (e.g., clinical systems are organized by accession numbers, where REMIX BI can be organized by any order desired).

While the foundation of REMIX is largely open-source in origin, customization has been needed to improve functionality; such enhancements will be made freely available although cannot be distributed along with software packages being sold commercially by a third party. Thus, end-users would need to acquire certain tools (e.g., Tableau, OBIEE) due to the absence of pre-existing special partnerships between our group and such vendors. The advantages of working with commercially available vendor-based tools are that as follows: (1) In general, they are well documented; (2) being commercially available, it is less likely that they will be discontinued; (3) support contracts are usually available; and (4) many are already purchased and are available within medical intuitions.

## Conclusion

We believe that the integrated capabilities developed as part of the REMIX platform will assist many clinicians, researchers, and administrators in optimizing fundamental imaging-related operations. While online sources for radiological data are available for research, for many institutions, datasets that are much larger exist within their own medical records. Whether data are extracted for institutional uses (e.g., quality assessment, education, or research), or whether datasets are to be extracted for multi-institutional or national projects, robust and flexible data management capabilities are essential.

### Acknowledgments and Funding Information

This project was partially funded by Edward J. DeBartolo, Jr. Family, and NIH Center for Accelerated Innovations.
